# Effect of national guidance on survival for babies born at 22 weeks’ gestation in England and Wales: population based cohort study

**DOI:** 10.1136/bmjmed-2023-000579

**Published:** 2023-11-07

**Authors:** Lucy K Smith, Emily van Blankenstein, Grenville Fox, Sarah E Seaton, Mario Martínez-Jiménez, Stavros Petrou, Cheryl Battersby, MBRRACE-UK Perinatal Surveillance Group

**Affiliations:** 1 Population Health Sciences, University of Leicester, Leicester, UK; 2 Neonatal Medicine, School of Public Health, Faculty of Medicine, Imperial College London, London, UK; 3 Neonatology, Guy’s and St Thomas’ NHS Foundation Trust, London, UK; 4 Department of Economics and Public Policy, Centre for Health Economics & Policy Innovation, Imperial College Business School, London, UK; 5 Nuffield Department of Primary Care and Health Science, Oxford University, Oxford, UK

**Keywords:** Neonatology, Obstetrics, Health services

## Abstract

**Objectives:**

To explore the effect of changes in national clinical recommendations in 2019 that extended provision of survival focused care to babies born at 22 weeks’ gestation in England and Wales.

**Design:**

Population based cohort study.

**Setting:**

England and Wales, comprising routine data for births and hospital records.

**Participants:**

Babies alive at the onset of care in labour at 22 weeks+0 days to 22 weeks+6 days and at 23 weeks+0 days to 24 weeks+6 days for comparison purposes between 1 January 2018 and 31 December 2021.

**Main outcome measures:**

Percentage of babies given survival focused care (active respiratory support after birth), admitted to neonatal care, and surviving to discharge in 2018-19 and 2020-21.

**Results:**

For the 1001 babies alive at the onset of labour at 22 weeks' gestation, a threefold increase was noted in: survival focused care provision from 11.3% to 38.4% (risk ratio 3.41 (95% confidence interval 2.61 to 4.45)); admissions to neonatal units from 7.4% to 28.1% (3.77 (2.70 to 5.27)), and survival to discharge from neonatal care from 2.5% to 8.2% (3.29 (1.78 to 6.09)). More babies of lower birth weight and early gestational age received survival focused care in 2020-21 than 2018-19 (46% to 64% at <500g weight; 19% to 31% at 22 weeks+0 days to 22 weeks+3 days).

**Conclusions:**

A change in national guidance to recommend a risk based approach was associated with a threefold increase in 22 weeks’ gestation babies receiving survival focused care. The number of babies being admitted to neonatal units and those surviving to discharge increased.

**WHAT IS ALREADY KNOW ON THIS TOPIC**

- International debate surrounds provision of selective versus universal survival focused care for babies born at 22 weeks’ gestation
- The proportion of babies surviving following birth at 22 weeks’ gestation varies internationally because of variations in approach to provision of survival focused care and no robust population denominators
- The effect on care provision of new national guidance in the UK, which recommends a risk based approach to survival focused care including babies from 22 weeks’ gestation, has not been evaluated

**WHAT THIS STUDY ADDS**

- Following changes to national guidance, babies born at 22 weeks’ gestation receiving survival focused care and being admitted to neonatal care increased threefold
- The number of babies being admitted to neonatal units and those surviving to discharge has increased following changes to practice

**HOW THIS STUDY MIGHT AFFECT RESEARCH, PRACTICE, OR POLICY**

- The findings have major implications for additional resource needs and are of vital importance for countries considering updating recommendations
- Identifying reliable factors that predict mortality, while considering complications that occur in the first few days or weeks after birth, might help to limit provision of intensive care support when survival is unlikely

## Introduction

Decisions around survival focused care of babies born at 22 weeks’ gestation are challenging^1 2^ and continue to spark debate about provision of survival focused care that is selective versus universal.^3^ Robust estimates of survival will help to inform national recommendations, individual decision making, and parental counselling. However, differences in the provision of survival focused care and the choice of denominator population used to calculate survival can affect or bias these calculations. Countries (eg, Netherlands and Denmark) where survival focused care at 22 weeks’ is not recommended, have extremely low reported levels of survival.^4 5^ By comparison, Japan, which has near universal survival focused care,^6^ reports higher proportions of survival (about 60%). However, caution is needed when interpreting results of small studies with selective populations across different healthcare systems. The use of live births as a denominator can lead to biased estimates of survival because interpretations of signs of life and classification of death varies between and within countries^7–10^ and because live birth registration may be strongly associated with positive prognostic factors for resuscitation.^8^ Using babies alive at the onset of care in labour (comprising intrapartum deaths and live births) as a denominator has been shown to be less susceptible to these biases.^8^ Furthermore, the perinatal care pathway for babies at risk of extreme preterm birth should begin with antenatal counselling involving discussions with parents to inform decision making.

Following a review of international survival data published online in 2019, the British Association of Perinatal Medicine developed a new consensus based framework for practice,^11^ updating guidelines from 2008.^12^ This new framework recommends that survival focused care may include babies from 22 weeks’ gestation following assessment of risk and discussion with parents (box 1). This guidance reflects similar changes in the United States^13^ and longer standing practices in Sweden^14^ and Japan.^6^

**Guidance on survival focused care for babies^11 12^**

**British Association of Perinatal Medicine, 2008**

If gestational age is certain and less than 23+0 (ie, at 22 weeks) for the best interests of the baby, and as per standard practice, babies should not be resuscitated. If the parents wish, they should have the opportunity to discuss outcomes with a second senior member of the perinatal team

**British Association of Perinatal Medicine, 2019**

Neonatal stabilisation may be considered for babies born from 22+0 weeks of gestation following assessment of risk and multidisciplinary professional discussion with parents. Attempts to resuscitate babies born before 22+0 weeks of gestation are not appropriate

We used this opportunity to evaluate the effect of this change in national guidance in England and Wales, which now recommends a risk based approach to survival focused care for babies born at 22 weeks’ gestation.^11^ We focused on admission to neonatal care and survival to discharge, with and without morbidities, to assess the implications for care provision and outcomes for babies.

## Methods

### Study design and data sets

We undertook a population based study using retrospective data from two national datasets in England and Wales: (1) mothers and babies: reducing risk through audits and confidential enquiries across the UK (MBRRACE-UK) and (2) the national neonatal research database (NNRD). These datasets both comprise complete coverage of England and Wales for the time period under study. MBRRACE-UK collects national perinatal mortality surveillance data with detailed information from UK hospitals on all UK deaths of babies from 22 weeks’ gestation, including fetal losses (22+0-23+6 weeks), stillbirths (≥24+0 weeks), and neonatal deaths (deaths ≤28 days),^15 16^ linked to birth notifications and birth, stillbirth, and death registrations to validate ascertainment. The NNRD^17^ contains data extracted from electronic health records^18^ for care and outcomes to neonatal discharge for all babies admitted to neonatal care within the National Health Service in England and Wales.^19^ We combined individual data into aggregated data tables by gestational age, time period, and each variable of interest from the two datasets: from MBRRACE-UK for babies who died in maternity care and from NNRD on babies surviving to admission to neonatal care, to obtain a full cohort of babies born at 22+0-24+6 weeks gestation.

### Study population

We included babies born (and whose mothers were resident) in England and Wales between 1 January 2018 and 31 December 2021 (inclusive) at 22+0 to 22+6 weeks’ gestational age. We also included data for births at 23+0-24+6 weeks for comparative purposes.

### Outcomes

The main outcomes were survival to admission for neonatal care, length of neonatal unit stay in days, survival to discharge from neonatal care (discharge home or to other healthcare settings, such as paediatric ward or intensive care unit), and survival to discharge without major morbidity. Major morbidity included: retinopathy of prematurity treated by use of laser or medication; severe brain injury (including seizures, moderate to severe hypoxic-ischaemic encephalopathy, stroke, grade three or four intracranial haemorrhage; central nervous system infection; kernicterus, cystic periventricular leukomalacia^20^); severe necrotising enterocolitis (confirmed at surgery); and bronchopulmonary dysplasia (defined as requirement for oxygen or respiratory support at 36 weeks postmenstrual age (also known as corrected gestational age, the sum of gestational age plus chronological age)). Due to near universal levels of bronchopulmonary dysplasia, a post-analysis decision was made to report survival with no major morbidity, excluding bronchopulmonary dysplasia from the major morbidity definition.

The main variable of interest was the provision of survival focused care, which was defined as provision of active respiratory support following birth. This component was used because it was available for all births whereas information on antenatal steroids and magnesium sulphate was only available for babies admitted to neonatal care. We compared data before and after the introduction of the new British Association of Perinatal Medicine framework for practice^11^ (published on 23 October 2019) using two time periods: 1 January 2018-31 December 2019 and 1 January 2020-31 December 2021, with time periods chosen to be the same length and include the same calendar periods.

We explored pregnancy and birth characteristics focusing on risk factors that the guidance from the British Association of Perinatal Medicine indicated should inform decision making and parental discussions. These comprised non-modifiable factors: gestational age (early (22+0-22+3), late (22+4-22+6)); fetal growth (categorised birth weight as <500 g, ≥500 g); sex (male, female); plurality (singleton, multiple). These risk factors also comprised modifiable factors: antenatal steroid exposure (available for babies admitted to neonatal care only: none, partial, or complete course) and setting for birth (out of hospital, care provider without *v* with tertiary neonatal intensive care unit). Additionally, we explored geographical region of birth (defined regions of the UK National Health Service), mode of delivery (unassisted *v* assisted vaginal birth, caesarean section or other), Apgar score at 1 min (≤1, >1), and provision of antenatal magnesium sulphate (available for babies admitted to neonatal care only).

### Statistical analysis

We present outcomes using four denominators: (1) babies alive at the onset of care in labour or onset of the birthing process (ie, vaginal births, births following caesarean section either live born or intrapartum stillbirths, and stillbirths of unknown timing, which were included in this denominator because the clinical approach during the birthing process was assumed to be similar to that for a known intrapartum stillbirth); (2) live births; (3) babies receiving survival focused care; and (4) babies admitted to neonatal care. For each reported outcomes, we calculated 95% confidence intervals (using the normal approximation). We report risk ratios with 95% confidence intervals to compare the change in percentages over the two time periods. Median (and interquartile range) length of stay and total care (days) are reported by gestation separately for babies who died and for those who survived to discharge. We also present survival based on hospital at birth categorised as hospitals with tertiary or non-tertiary neonatal units or out of hospital. Data were compared for two 24 month time periods approximately before and after the implementation of the new guidance: 1 January 2018-31 December 2019 and 1 January 2020-31 December 2021. Specifically, we used a single group interrupted time series analysis that was estimated by a two stage least-squares regression allowing for the variability to be accounted for during the estimation.^21^ An interrupted time series was an appropriate quasi-experimental design for this analysis because a comparison group was not needed^22^ and the design accounted for the seasonal elements present in the monthly level dataset.^21^ The model operated under the assumption that any time varying confounder was relatively slowly changing so that the confounder was indistinguishable from the change in trend caused by the intervention. We accounted for complex correlation structures in the residuals by estimating robust standard errors.

To assess the effect of the British Association of Perinatal Medicine guidance on survival focused care, we undertook an interrupted time series analysis. We calculated estimates and confidence intervals for the increase in the trend over time from the baseline period before guidance introduction (1 January 2018-31 December 2019) compared with the guidance consultation period (1 June 2019-31 October 2019), and following publication of the guidance (1 November 2019-31 December 2021).

### Sensitivity analyses

We compared the alignment of the NNRD and MBRRACE-UK datasets by analysing the number of births common to both datasets (ie, babies alive at the onset of care in labour, admitted to neonatal care, and died within 28 days of birth) by year and gestational age.

### Patient and public involvement

Patients and the public were not involved in the design, or conduct, or reporting, or dissemination plans of this research as this study was evaluating the clinical impact of a change in guideline with no active research recruitment; however, future research in this area will embed patient and public involvement throughout.

## Results

### Number of babies alive at the onset of labour

Overall, 5623 babies were born at 22+0-24+6 weeks’ gestation including 1604 antepartum stillbirths. Of the 4019 babies alive at the onset of care in labour (including 178 stillbirths of unknown timing), 1001 (25%) were at 22 weeks gestational age, 1380 (34%) were at 23 weeks, and 1638 (41%) were at 24 weeks. Between the two time periods (2018-19 *v* 2020-21), the number of babies alive at the onset of care in labour reduced from 524 to 477 at 22 weeks (representing a 9% decrease in absolute numbers of births), from 725 to 655 at 23 weeks (10% decrease), and from 887 to 751 births at 24 weeks (15% decrease) (table 1).

**Table 1:** Number and percentage of babies alive at the onset of care in labour, receiving survival focused care, admitted to neonatal care, and surviving to discharge from neonatal care by gestational age in weeks and days and by year of birth (2018-19 *v* 2020-21)

Population	Gestational age of 22+0-22+6		Gestational age of 23+0-23+6		Gestational age of 24+0-24+6	
	2018-19	2020-21	2018-19	2020-21	2018-19	2020-21
All births (n)	846	727	995	892	1170	993
Babies alive at onset of labour (n)	524	477	725	655	887	751
Live births (n)	295	319	617	559	809	700
Babies receiving survival focused care (n):	59	183	576	528	814	700
%, babies alive at onset of labour (95% CI)	11.3 (8.6 to 14)	38.4 (34 to 42.7)	79.4 (76.5 to 82.4)	80.6 (77.6 to 83.6)	91.8 (90 to 93.6)	93.2 (91.4 to 95)
%, live births (95% CI)	20.0 (15.4 to 24.6)	57.4 (51.9 to 62.8)	93.4 (91.4 to 95.3)	94.4 (92.6 to 96.4)	100.6* (N/A)	100.0 (100 to 100)
Babies admitted to neonatal care (n):	39	134	504	467	768	669
%, babies alive at onset of labour (95% CI)	7.4 (5.2 to 9.7)	28.1 (24.1 to 32.1)	69.5 (66.2 to 72.9)	71.3 (67.8 to 74.8)	86.6 (84.3 to 88.8)	89.1 (86.9 to 91.3)
%, live births (95% CI)	13.2 (9.4 to 17.1)	42 (36.6 to 47.4)	81.7 (78.6 to 84.7)	83.5 (80.5 to 86.6)	94.9 (93.4 to 96.4)	95.6 (94 to 97.1)
%, babies receiving survival focused care (95% CI)	66.1 (54 to 78.2)	73.2 (66.8 to 79.6)	87.5 (84.8 to 90.2)	88.4 (85.7 to 91.2)	94.3 (92.8 to 95.9)	95.6 (94 to 97.1)
Babies surviving to discharge from neonatal care (n):	13	39	231†	222†	518	483†
%, babies alive at onset of labour (95% CI)	2.5 (1.1 to 3.8)	8.2 (5.7 to 10.6)	31.9 (28.5 to 35.3)	33.9 (30.3 to 37.5)	58.4 (55.2 to 61.6)	64.3 (60.9 to 67.7)
%, live births (95% CI)	4.4 (2.1 to 6.7)	12.2 (8.6 to 15.8)	37.4 (33.6 to 41.3)	39.7 (35.7 to 43.8)	64 (60.7 to 67.3)	69.0 (65.6 to 72.4)
%, babies receiving survival focused care (95% CI)	22.0 (11.5 to 32.6)	21.3 (15.4 to 27.2)	40.1 (36.1 to 44.1)	42.0 (37.8 to 46.3)	63.6 (60.3 to 66.9)	69.0 (65.6 to 72.4)
%, babies admitted to neonatal care (95% CI)	33.3 (18.5 to 48.1)	29.1 (21.4 to 36.8)	45.8 (41.5 to 50.2)	47.5 (43.0 to 52.1)	67.4 (64.1 to 70.8)	72.2 (68.8 to 75.6)
Babies surviving to discharge without morbidities¶ (n):	0	14	95†	72†	233	230†
%, babies alive at onset of labour (95% CI)	—§	2.9 (1.4 to 4.5)	13.1 (10.7 to 15.6)	11.0 (8.6 to 13.4)	26.3 (23.2 to 29.4)	30.6 (27.3 to 33.9)
%, live births (95% CI)	—§	4.4 (2.1 to 6.6)	15.4 (12.6 to 18.2)	12.9 (10.1 to 15.7)	28.8 (25.7 to 31.9)	32.9 (29.4 to 36.3)
%, babies receiving survival focused care (95% CI)	—§	7.7 (3.8 to 11.5)	16.5 (13.5 to 19.5)	13.6 (10.7 to 16.6)	28.6 (25.4 to 31.8)	32.9 (29.4 to 36.3)
%, babies admitted to neonatal care (95% CI)	—§	10.4 (5.3 to 15.6)	18.8 (15.4 to 22.3)	15.4 (12.1 to 18.7)	30.3 (27.1 to 33.6)	34.4 (30.8 to 38.0)
Babies surviving to discharge without morbidities including BPD¶ (n)	0	0	0	0	28†	32†

For percentages, the denominator is detailed in the table.

BPD=bronchopulmonary dysplasia; CI=confidence interval; N/A=not available; NEC=necrotising enterocolitis; ROP=retinopathy of prematurity.

*Percentage >100 due to inclusion of babies who had an attempt of resuscitation but the death was classified as an intrapartum stillbirth.

†At 23 weeks, in 2018-19, one missing outcome data; in 2020-21, two missing outcome data and three remained in neonatal care at the time of data download; at 24 weeks, in 2018-19, two missing outcome data for BPD due to discharge before 36 weeks; in 2020-21, seven missing outcome data.

‡ROP, severe brain injury, or severe NEC

§Data masked due to small numbers.

¶Survival without BPD, ROP, severe brain injury, or severe NEC

### Survival focused care as a proportion of babies alive at the onset of labour

**Figure 1:**
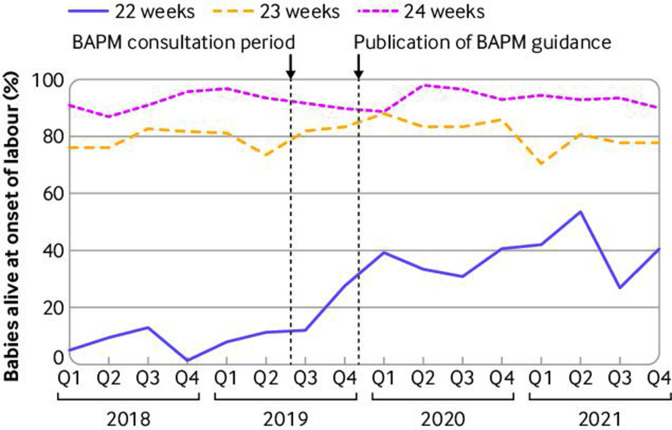
Babies who received survival focused care (as a percentage of all babies alive at the onset of care in labour) by gestational age in weeks and date of birth (calculated on a quarterly basis). BAPM=British Association of Perinatal Medicine

Of the babies who were alive at the onset of care in labour at 22 weeks, the number and percentage receiving survival focused care increased threefold from 59 (11%) in 2018-19 to 183 (38%) in 2020-21 (table 1, figure 1; risk ratio 3.41 (95% confidence interval 2.61 to 4.45)). By contrast, survival focused care estimates were consistently high from 2018 to 2021 at 23 weeks gestation (79% *v* 81%, risk ratio 1.01 (95% confidence interval 0.96 to 1.07)) and 24 weeks gestation (92% *v* 93%, 1.02 (0.99 to 1.04)). For births at 22 weeks, the interrupted time series analysis showed a relatively constant percentage of babies provided with survival focused care prior to the publication of the British Association of Perinatal Medicine guidance followed by a statistically significant change in the outcome variable immediately following the introduction of guidance and this change was sustained over time (table 2, figure 1). The percentage of babies provided with survival focused care increased by 17.8 per 100 births (95% confidence interval 9.4% to 26.2%) in the guidance consultation period (1 June 2019-31 October 2019) and by 30.7% per 100 births (14.6% to 46.9%) following publication of the guidance (1 November 2019-31 December 2021), compared with the period before guidance introduction (1 January 2018-31 May 2019). Additionally, provision of survival focused care increased 5.4% per month (95% confidence interval 0.2% to 10.6%) after the consultation and 5.9% per month (0.6% to 11.1%) after the publication of the guidance. No change was noted for babies born at 23-24 weeks either in the survival across the different time periods or in the trend over time (table 2).

**Table 2:** Parameter estimates (95% confidence interval) of interrupted time series analysis for proportion of babies alive at the onset of care in labour receiving survival focused care by gestational age of birth

Interrupted time series analysis	22 weeks’ gestation	23 weeks’ gestation	24 weeks’ gestation
Baseline proportion, Jan 2018 to Dec 2021	0.078 (0.025 to 0.131)	0.776 (0.726 to 0.826)	0.881 (0.832 to 0.929)
Increase in baseline, Jun 2019 to Oct 2019	0.178 (0.094 to 0.262)	0.050 (−0.035 to 0.137)	0.062 (0.099 to −0.025)
Increase in baseline, Nov 2019 to Dec 2021	0.307 (0.146 to 0.469)	0.013 (−0.110 to 0.136)	0.019 (−0.030 to 0.070)
Baseline trend over time, Jan 2018 to Dec 2021	0.001 (−0.005 to 0.002)	0.001 (−0.003 to 0.006)	0.005 (0 .001 to 0.009)
Increase in baseline time trend, Jun 2019 to Oct 2019	0.054 (0.002 to 0.106)	0.022 (−0.020 to 0.064)	−0.007 (−0.022 to 0.008)
Increase in baseline time trend, Nov 2019 to Dec 2021	0.059 (0.006 to 0.111)	−0.027 (−0.071 to 0.016)	0.001 (−0.012 to 0.015)

Dates represent the first of the month to the last of the month of those specified. The Cumby-Huizinga test for serial auto-correlation does not provide strong enough evidence to suggest that the serial correlation observed is inconsistent with the percentage of births provided with survival focused care being dependent on current and past error terms (a moving average process).

### Characteristics of babies who received survival focused care

**Figure 2:**
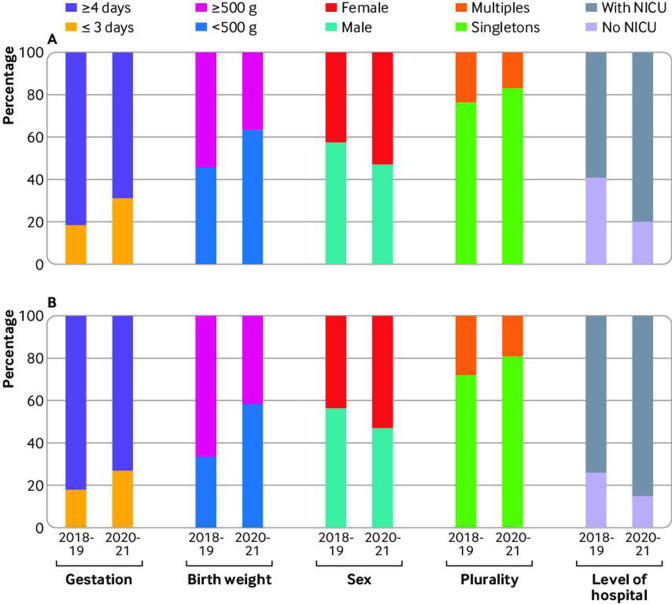
Percentage of 22 week babies by birth characteristics for each time period 2018-19 and 2020-21 for (A) babies receiving survival focused care and (B) babies admitted to neonatal care. NICU=neonatal intensive care unit

Provision of survival focused care for babies alive at the onset of care in labour at 22 weeks increased from 2018-19 to 2020-21 for all levels of risk factors defined as non-modifiable by the British Association of Perinatal Medicine guidance (table 3).^11^ Survival focused care rose from 4% to 23% for those born at 22+0-22+3 weeks’ gestation compared with a rise from 20% to 54% for those born at 22+4-22+6 weeks’ gestation. Similarly, care provision focused on survival increased for babies with a birth weight of less than 500 g (8% to 35%) and also for those weighing 500 g and more (20% to 48%). Survival focused care increased more for female babies (11% to 46%) than for male babies (12% to 34%); similar increases were noted for multiple gestation births (11% to 35%) and singleton births (11% to 39%). Consequently, the population of babies receiving survival focused care changed over time with an increase in smaller, more immature babies (46% to 64% for <500 g, 19% to 31% for 22+0-22+3 days), a reduction in the proportion of male babies (58% to 47%) and multiple births (24% to 17%), (figure 2) and a reduction in babies with an Apgar score of more than 1 (82% to 72%).

**Table 3:** Number of babies at 22 weeks’ gestation who were alive at onset of care in labour and percentage (number) of babies who received survival focused care and were admitted for neonatal care by birth characteristics for 2018-19 versus 2020-21 (% based on babies alive at onset of labour)

Outcome	No. alive at onset of labour		% Babies alive at onset of labour receiving survival focused care (No.)		% Babies alive at onset of labour admitted to neonatal care (No.)	
	2018-19	2020-21	2018-19	2020-21	2018-19	2020-21
Gestation (days) (missing n=9):						
≤3	276	244	4 (11)	23.4 (57)	2.5 (7)	14.8 (36)
≥4	240	232	20 (48)	53.9 (125)	13.3 (32)	42.2 (98)
Birth weight (missing n=32):						
<500 g	341	328	7.9 (27)	35.1 (115)	3.8 (13)	23.8 (78)
≥500 g	163	137	19.6 (32)	48.2 (66)	16 (26)	40.9 (56)
Sex (missing n=34):						
Male	282	256	12.1 (34)	33.6 (86)	7.8 (22)	24.6 (63)
Female	222	207	11.3 (25)	46.4 (96)	7.7 (17)	34.3 (71)
Plurality (missing n=0):						
Singleton	397	389	11.3 (45)	39.1 (152)	7.1 (28)	27.8 (108)
Multiple	127	88	11 (14)	35.2 (31)	8.7 (11)	29.5 (26)
Place of hospital (missing n=0):						
Out of hospital	24	19	37.5 (9)	47.4 (9)	*–	*–
Non-tertiary hospital	260	164	5.8 (15)	17.1 (28)	2.3 (6)	10.4 (17)
Tertiary hospital	240	294	14.6 (35)	49.7 (146)	12.1 (29)	38.8 (114)
Region (missing n=7):						
London	111	97	6.3 (7)	33 (32)	4.5 (5)	27.8 (27)
Midlands and east	119	108	13.4 (16)	42.6 (46)	8.4 (10)	30.6 (33)
North	129	125	14 (18)	41.6 (52)	10.1 (13)	25.6 (32)
South	146	121	8.2 (12)	35.5 (43)	4.1 (6)	26.4 (32)
Wales	13	23	*–	30.4 (7)	*–	30.4 (7)
Mode of delivery (missing n=7):						
Unassisted vaginal	479	422	11.1 (53)	38.2 (161)	7.5 (36)	28.7 (121)
Assisted vaginal	38	37	*–	21.6 (8)	*–	*–
Caesarean section or other	5	13	*–	76.9 (10)	*–	61.5 (8)
Apgar score (missing n=335):						
≤1	228	177	3.1 (7)	22.6 (40)	*–	10.7 (19)
≥2	120	141	26.7 (32)	72.3 (102)	23.3 (28)	62.4 (88)
Antenatal steroid provision (missing n=4):						
Yes	N/A	N/A	N/A	N/A	59.0 (23)	76.2 (99)
No	N/A	N/A	N/A	N/A	41.0 (16)	23.8 (31)
Magnesium sulphate provision (missing n=2):						
Yes	N/A	N/A	N/A	N/A	55.3 (21)	69.9 (93)
No	N/A	N/A	N/A	N/A	44.7 (17)	30.1 (40)

*Data masked due to small numbers. Data for antenatal steroids and magnesium sulphate includes complete and incomplete antenatal steroid courses.

The number of babies alive at the onset of care in labour who were born in a hospital with a tertiary neonatal intensive care unit increased from 240 to 294 (46% to 62%). Similar relative increases in survival focused care were noted for babies born in a hospital with or without a tertiary neonatal intensive care unit and across geographical regions although rates varied between regions (table 3). More than 90% of births were unassisted vaginal births. Although the number of caesarean sections increased, numbers were small (5 *v* 13, table 3).

Neonatal unit admissions as a proportion of babies alive at the onset of care in labour increased nearly fourfold from 39 (7%) to 134 (28%) (risk ratio 3.77 (95% confidence interval 2.70 to 5.27)), and as a proportion of those who received survival focused care increased slightly from 66% to 73% (table 1). Maternal magnesium sulphate for neonatal admissions increased from 55% to 70% and antenatal steroids from 59% to 76% (table 3).

### Outcomes

**Figure 3:**
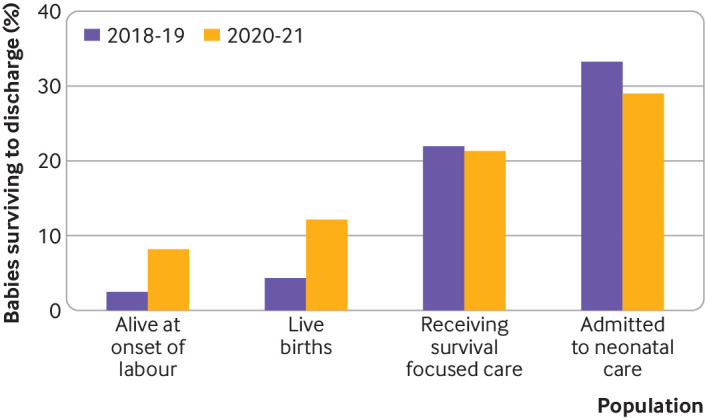
Percentage of babies surviving to discharge by time period for babies who were alive at the onset of care in labour, live births, babies receiving survival focused care and babies admitted to neonatal care

The absolute number of babies born at 22 weeks’ gestation surviving to discharge from neonatal care increased threefold between the two periods from 13 to 39 (table 1). Figure 3 illustrates survival using four denominators: (1) babies alive at the onset of care in labour, 2.5% versus 8.2% (risk ratio 3.30 (95% confidence interval 1.78 to 6.10)); (2) live births, 4% versus 12% (2.77 (1.51 to 5.09)); (3) babies receiving survival focused care, 22% versus 21% (0.97 (0.56 to 1.68)); and (4) babies admitted to neonatal care 33% versus 29% (0.87 (0.52 to 1.46)). The absolute numbers of babies admitted to neonatal units who died before discharge also increased from 26 to 95.

Provision of survival focused care and survival to discharge from neonatal care was higher for both time periods for babies born at 22 weeks’ gestation in a tertiary versus non-tertiary hospital for babies alive at the onset of care in labour (table 3). No babies survived if they were not born in a hospital with a neonatal unit, regardless of whether survival focused care was provided, although overall numbers were small.

Over the two time periods combined, survival of babies given survival focused care was 11.6% (5/43) for birth in non-tertiary hospitals versus 26.0% (47/181) for birth in tertiary hospitals. Almost all babies born at 22-24 weeks gestation and who survived to 36 weeks postmenstrual age had a requirement for oxygen or respiratory support at 36 weeks postmenstrual age. Additionally, we report survival to discharge without morbidity excluding bronchopulmonary dysplasia (table 1). For 2018-19, numbers were below the threshold for disclosure control at 22 weeks’ gestation and cannot be reported.^21^ Of babies who survived and were born in 2020-21, 36% (14/39) did not have a major morbidity (excluding bronchopulmonary dysplasia). Corresponding figures at 23 weeks were 32% (72/222) and at 24 weeks were 47% (230/483).

The total care days provided to all babies born at 22 weeks’ gestation was 2535 days in 2018-19 (1268 per annum) and 6840 days in 2020-21 (3420 per annum) (online supplemental table S1). The median number of care days for babies born at 22 weeks’ gestation and died was 3 (interquartile range 1-4) in 2018-19 versus 4 (2-11) in 2020-21, and for those who survived was 153 (142-160) versus 154 (132-169).

10.1136/bmjmed-2023-000579.supp1 Supplementary data

### Sensitivity analyses

Validation of the alignment of the NNRD and MBRRACE-UK datasets showed similar numbers of babies at 22+0–24+6 weeks born alive at the onset of care in labour, admitted to neonatal care, but died in the neonatal period (common to both datasets) (MBRRACE-UK, n=887; NNRD, n=865). This difference of 2.5% was similar by year and gestational age; this mall proportion had a negligible effect on survival estimates.

## Discussion

We explored the effect of a change in national guidance around the new recommendation of a risk based approach to the provision of survival focused care on outcomes for babies born at 22 weeks’ gestation in England and Wales. We found a threefold increase in the number of babies receiving survival focused care (59-183 babies) and being admitted to neonatal care (39-134 babies) after publication of the guidance. Our analysis suggests that these rapid and substantial changes were associated with the introduction of the British Association of Perinatal Medicine guidance. Although the recommendation was intended to be risk based, we speculate that, on the contrary, approaches have moved from being selective to more widespread provisions of survival focused care. This change would explain the increase in the proportion of babies at high risk who received survival focused care. For example, where previously most babies receiving survival focused care weighed 500 g and more, care for babies weighing under 500 g now outnumbers that for babies weighing 500 g and more.

Despite our finding of an improvement in perinatal optimisation practice (provision of antenatal steroids, magnesium sulphate, and births in a hospital with a tertiary neonatal intensive care unit), and an increased number of babies who survived, survival overall did not improve. Although this result is not unexpected, because improvements in survival may only be noted as expertise grows in the care of these babies, we speculate that this finding may partly be due to differences in population characteristics. However, the numbers are too small to detect differences and should be interpreted with caution. The increase in the percentage of babies provided with survival focused care and being admitted to neonatal care led to nearly a threefold increase in duration of total neonatal care days for babies born at 22 weeks’. These findings reflect the substantial impact of these changes on activity and occupancy levels, as well as clinical complexity. Low rates of survival despite prolonged periods of intensive care can be ethically and emotionally challenging for families and health care professionals. The effect of survival focused care on survival may take time to evaluate as expertise grows in the care of these babies. However, in the absence of evidence based prognostic factors in this new population of babies, intensive care support provides time to assess response, with an opportunity to reorientate intensive care support if that option is not in the best interest of the baby or where survival is unlikely. Future research is needed to understand the perspectives of parents, healthcare professionals, and wider society on the effects of such a change in practice. Additionally research on long term follow-up of these children is needed to determine neurodevelopmental outcomes and the implications for parents and healthcare and educational systems.

These latest findings using appropriate robust denominators will help to inform service needs and parental counselling at key time points in the perinatal pathway. The findings may be presented as: four in 10 babies alive at the onset of care in labour will be expected to receive survival focused care; two in 10 babies who receive survival focused care and three in 10 admitted to neonatal care would be expected to survive to discharge (ie, seven in 10 die); and one in 10 babies admitted to neonatal care are expected to survive to discharge without major morbidities (excluding bronchopulmonary dysplasia), although their long term outcomes are not known. The near universal prevalence of bronchopulmonary dysplasia among babies born at 22-24 weeks’ gestation was not surprising, and raises the question of whether bronchopulmonary dysplasia is a discriminatory outcome measure in this clinical population.^23^

### Comparison with other studies

Although we have highlighted that survival estimates based on live births may be less robust, for comparison to international figures, we present these data, with increases in: the percentage of babies being given survival focused care from 20% to 57%, babies being admitted to neonatal care from 13% to 42%, and babies surviving to discharge from 4% to 12%. This threefold increase in survival outcomes of live births over time are similar to those reported elsewhere where provision of survival focused care for babies born at 22 weeks’ has been introduced.^1 24^ Our estimates of survival to discharge in 2020 to 2021 were slightly higher than a meta-analysis published in 2019, with 12% versus 4% for live births at 22 weeks’ and 29% versus 24% for admissions to neonatal care 25 . Our survival estimates following survival focused care were much lower than those reported for Japan (>60%), which introduced universal provision of survival focused care for babies born at 22 weeks gestation over 10 years ago.^6 26^ However, we have highlighted the bias that may be introduced by using live births as the population denominator,^27^ as was used for the estimates in Japan, which makes international comparisons unreliable in contrast to using the denominator alive at the onset of care in labour that we focus our analyses on.^8^ Our finding of higher levels of survival for babies born at 22 weeks’ gestation in a tertiary hospital is consistent with previously published outcomes for all babies born extremely preterm.^28^

### Strengths and limitations

A key strength of our study is the use of robust denominators to evaluate the impact of a more widespread approach to survival focused care of babies at 22 weeks’ gestational age. In the more recent time period of 2020-21, a higher proportion of births at 22 weeks’ were reported as live births,^29^ likely influenced by the lowering of the gestational age threshold of survival.^30^ This further emphasises the importance of use of babies alive at the onset of care in labour as the denominator. We were able to have population coverage by combining data from two national datasets, NNRD and MBRRACE-UK, which closely aligned in definitions and numbers. Other studies have compared different approaches to survival focused care across different geographical populations,^31^ whereas our analyses allowed the assessment of the effect of the changes on a consistent national population.

We acknowledge study limitations. Our definition of survival focused care was mainly about provision of active respiratory care because this information was uniformly available for the whole cohort of births and data for respiratory care was only missing for 16 (0.5%) out of 3299 live births. We recognised that survival focused care at these extremely low gestational ages is a multidisciplinary approach across both obstetrics and neonatology, including the provision of antenatal steroids and magnesium sulphate. We, therefore, may have underestimated the provision of all types of survival focused care. Although we had access to monthly data, due to the small numbers, we aggregated data into two equal time periods closely aligned with the guidance from the British Association of Perinatal Medicine for our primary analysis. Comparisons of survival without major morbidity between time periods is limited due to small numbers and no individualised linked data, which prevented exploration of multiple regression models that allow for more detailed understanding of the effect of birth characteristics. Although our definition of survival focused care was restricted to provision of respiratory support following birth, this outcome provides a good proxy for a range of survival focused factors and we await evidence from the ongoing TRANSFER study,^32^ which aims to assess the incidence of at risk preterm birth in women presenting at 22+0-23+6 weeks’ gestation. Also, we were only able to report short term outcomes to neonatal discharge. Future data linkage in the neoWONDER study will link these data to longer term health, education, and resource use outcomes.^33^ Furthermore, whereas here we were unable to explore individual level data, our future planned work includes an exploration of resource and cost implications and factors that affect survival, using multivariable analyses. We recognise that the introduction of the British Association of Perinatal Medicine guidance may have coincided with other factors that affect the outcomes that we have measured. However, similar to other reported studies, we found a decrease in births of extremely preterm babies in 2020, concurrent with the covid-19 pandemic and associated national lockdown.^34^ Although we expect this effect to have impacted the absolute number of births at 22-24 weeks, we do not expect this decrease to have influenced the proportion of babies provided with survival focused care.

These findings are of key relevance for countries considering similar changes to national recommendations because potential impacts can affect babies, families, healthcare professionals, and the healthcare system. International collaboration is continually needed to bring together clinicians and researchers worldwide; to learn from each other and improve the care of babies born at 22 weeks’ gestation to improve morbidity-free survival. The increased use of standardised robust denominators for the calculation of survival estimates, such as babies alive at the onset of care in labour, is a key part of ensuring increased comparability of international findings that can aid future improvements.

## Conclusions

While survival for babies born at 22 weeks remains low, the numbers receiving survival focused care and being admitted to neonatal units has increased tripled. Although this finding suggests that the total number of survivors has increased, this result also means that the number of babies who died after intensive care also increased. Maternity care was also affected because of likely increases in in-utero transfers (ie, moved to a specialist hospital before birth), as well as impacts on paediatric and educational services to provide for long term health and developmental needs. This change represents an important increase in workload and need for specialised health care and educational resources.

As clinical experience caring for this vulnerable group of babies grows, a research priority is to identify reliable prognostic factors in the first few days and weeks so that prolonged intensive care can be avoided.

## Data Availability

Data may be obtained from a third party and are not publicly available. MBRRACE-UK data may be requested from the data controller, the healthcare quality improvement partnership. A data access request form can be obtained from https://www.hqip.org.uk/national-programmes/accessing-ncapop-data/%23.XQeml_lKhjU national neonatal research database data are available on reasonable request. All data relevant to the study are included in the article or uploaded as supplementary information. The national neonatal research database is a national data asset discoverable through the health data research UK alliance innovation gateway (http://www.healthdatagateway.org/) and is available for use by external investigators.

## References

[1] Rysavy MA, Mehler K, Oberthür A, et al. An immature science: intensive care for infants born at ≤23 weeks of gestation. J Pediatr 2021;233:16–25. 10.1016/j.jpeds.2021.03.006 33691163 PMC8154715

[2] Lantos JD. Ethics of care for the micropreemies. Just because we can, should we? Semin Fetal Neonatal Med 2022;27:101343. 10.1016/j.siny.2022.101343 35514009

[3] Lee CD, Nelin L, Foglia EE. Neonatal resuscitation in 22-week pregnancies. N Engl J Med 2022;386:391–3. 10.1056/NEJMclde2114954 35081286

[4] Wilkinson D, Verhagen E, Johansson S. Thresholds for resuscitation of extremely preterm infants in the UK, Sweden, and Netherlands. Pediatrics 2018;142:S574–84. 10.1542/peds.2018-0478I 30171144 PMC6379058

[5] Lantos JD, Carter B, Garrett J. Do sociocultural factors influence periviability counseling and treatment more than science? Lessons from Scandinavia. Pediatrics 2018;142:S600–2. 10.1542/peds.2018-0478M 30171148

[6] Isayama T. The clinical management and outcomes of extremely preterm infants in Japan: past, present, and future. Transl Pediatr 2019;8:199–211. 10.21037/tp.2019.07.10 31413954 PMC6675688

[7] Kc A, Berkelhamer S, Gurung R, et al. The burden of and factors associated with misclassification of intrapartum stillbirth: evidence from a large scale multicentric observational study. Acta Obstet Gynecol Scand 2020;99:303–11. 10.1111/aogs.13746 31600823

[8] Smith LK, Morisaki N, Morken N-H, et al. An international comparison of death classification at 22 to 25 weeks' gestational age. Pediatrics 2018;142:e20173324. 10.1542/peds.2017-3324 29899042

[9] Smith L, Draper ES, Manktelow BN, et al. Comparing regional infant death rates: the influence of preterm births less than 24 weeks of gestation. Arch Dis Child Fetal Neonatal Ed 2013;98:F103–7. 10.1136/fetalneonatal-2011-301359 22684158 PMC3582045

[10] Joseph KS, Liu S, Rouleau J, et al. Influence of definition based versus pragmatic birth registration on international comparisons of perinatal and infant mortality: population based retrospective study. BMJ 2012;344:e746. 10.1136/bmj.e746 22344455 PMC3281499

[11] Mactier H, Bates SE, Johnston T, et al. Perinatal management of extreme preterm birth before 27 weeks of gestation: a framework for practice. Arch Dis Child Fetal Neonatal Ed 2020;105:232–9. 10.1136/archdischild-2019-318402 31980443

[12] Wilkinson AR, Ahluwalia J, Cole A, et al. Management of babies born extremely preterm at less than 26 weeks of gestation: a framework for clinical practice at the time of birth. Arch Dis Child Fetal Neonatal Ed 2009;94:F2–5. 10.1136/adc.2008.143321 18838468

[13] Cummings J, Committee on Fetus and Newborn. Antenatal counseling regarding resuscitation and intensive care before 25 weeks of gestation. Pediatrics 2015;136:588–95. 10.1542/peds.2015-2336 26324869

[14] Domellöf M, Pettersson K. Guidelines for threatening premature birth will provide better and more equal care. Lakartidningen 2017;114:EEYI. 28463389

[15] Draper ES, Gallimore ID, Smith LK, et al. MBRRACE-UK perinatal mortality surveillance report, UK perinatal deaths for births from January to December 2017; 2019.

[16] MBRRACE-UK. Available: https://www.npeu.ox.ac.uk/mbrrace-uk

[17] Battersby C, Statnikov Y, Santhakumaran S, et al. The United Kingdom National Neonatal Research Database: a validation study. PLoS ONE 2018;13:e0201815. 10.1371/journal.pone.0201815 30114277 PMC6095506

[18] DAPB1595: Neonatal Data Set, Available: https://digital.nhs.uk/data-and-information/information-standards/information-standards-and-data-collections-including-extractions/publications-and-notifications/standards-and-collections/dapb1595-neonatal-data-set

[19] National neonatal research database. Available: https://www.imperial.ac.uk/neonatal-data-analysis-unit/neonatal-data-analysis-unit/contributing-to-the-national-neonatal-research-database/

[20] Gale C, Statnikov Y, Jawad S, et al. Neonatal brain injuries in England: population-based incidence derived from routinely recorded clinical data held in the national neonatal research database. Arch Dis Child Fetal Neonatal Ed 2018;103:F301–6. 10.1136/archdischild-2017-313707 29180541 PMC6047140

[21] Linden A. Conducting interrupted time-series analysis for single- and multiple-group comparisons. Stata J 2015;15:480–500. 10.1177/1536867X1501500208

[22] Fretheim A, Soumerai SB, Zhang F, et al. Interrupted time-series analysis yielded an effect estimate concordant with the cluster-randomized controlled trial result. J Clin Epidemiol 2013;66:883–7. 10.1016/j.jclinepi.2013.03.016 23810027

[23] Barrington KJ, Church PT, Luu TM, et al. Respiratory outcomes in preterm babies: is bronchopulmonary dysplasia important? Acta Paediatr 2022;111:1660–3. 10.1111/apa.16417 35608213

[24] Norman M, Hallberg B, Abrahamsson T, et al. Association between year of birth and 1-year survival among extremely preterm infants in Sweden during 2004–2007 and 2014–2016. Obstet Gynecol Surv 2019;74:456–8. 10.1097/01.ogx.0000577812.20743.bf PMC6439685 30912837

[25] Myrhaug HT, Brurberg KG, Hov L, et al. Survival and impairment of extremely premature infants: a meta-analysis. Pediatrics 2019;143. 10.1542/peds.2018-0933 30705140

[26] Nishida H, Ishizuka Y. Survival rate of extremely low birthweight infants and its effect on the amendment of the eugenic protection act in Japan. Acta Paediatr Jpn 1992;34:612–6. 10.1111/j.1442-200x.1992.tb01020.x 1285508

[27] Smith LK, Blondel B, Zeitlin J, et al. Producing valid Statistics when legislation, culture and medical practices differ for births at or before the threshold of survival: report of a European workshop. BJOG 2020;127:314–8. 10.1111/1471-0528.15971 31580509 PMC7003918

[28] Marlow N, Bennett C, Draper ES, et al. Perinatal outcomes for extremely preterm babies in relation to place of birth in England: the epicure 2 study. Obstet Gynecol Surv 2014;69:577–9. 10.1097/01.ogx.0000456347.54969.f4 PMC3995269 24604108

[29] Nath S, Hardelid P, Zylbersztejn A. Are infant mortality rates increasing in England? The effect of extreme prematurity and early neonatal deaths. J Public Health (Oxf) 2021;43:541–50. 10.1093/pubmed/fdaa025 32119086 PMC8458015

[30] Mercer BM. Periviable birth and the shifting limit of viability. Clin Perinatol 2017;44:283–6. 10.1016/j.clp.2017.02.002 28477660

[31] Backes CH, Söderström F, Ågren J, et al. Outcomes following a comprehensive versus a selective approach for infants born at 22 weeks of gestation. J Perinatol 2019;39:39–47. 10.1038/s41372-018-0248-y 30353079

[32] Transfer study. Available: https://www.birmingham.ac.uk/documents/college-mds/trials/bistc/transfer-protocol-v3.0-28-oct-21.pdf

[33] Neowonderstudy. Available: www.neowonder.org.uk

[34] Greenbury SF, Longford N, Ougham K, et al. Changes in neonatal admissions, care processes and outcomes in England and Wales during the COVID-19 pandemic: a whole population cohort study. BMJ Open 2021;11:e054410. 10.1136/bmjopen-2021-054410 PMC8488283 34598993

