# Correction: *Effect of national guidance on survival for babies born at 22 weeks’ gestation in England and Wales: population based cohort study*

**DOI:** 10.1136/bmjmed-2023-000579corr1

**Published:** 2023-11-15

In this research article by Smith and colleagues (*BMJMED* 2023;**2**:1–11. doi: 10.1136/bmj-2023-000579, published 7 Nov), the axes in figure 3 and the headings in table 3 have been updated.

