# Supplementary material for: Effect of national guidance on survival for babies born at 22 weeks’ gestation in England and Wales: population based cohort study
Source: BMJ Med. 2023 Nov 7;2(1):e000579. doi: 10.1136/bmjmed-2023-000579 (PMC10649719; doi:10.1136/bmjmed-2023-000579)
Supplement: Supplementary data [file bmjmed-2023-000579supp001.pdf]

Supplementary table S1: Median duration of neonatal admission in days by gestational age, time period and survival status for births at 22^{+0} to 24^{+6} weeks gestational age

Gestation (weeks)	22				23				24			
Time period	2018-2019		2020-2021		2018-2019		2020-2021		2018-2019		2020-2021	
Survival	Died	Survived	Died	Survived	Died	Survived	Died	Survived	Died	Survived	Died	Survived
Median length of admission in days (Interquartile range)	3 (1 – 14)	153 (142 – 160)	4 (2 – 11)	154 (132 – 169)	7 (2 – 17)	135 (116 – 155)	6 (2 – 18)	136 (120 – 159)	9 (3 – 29)	120 (105 – 141)	10 (4 –27)	119 (104-137)
Total care days	497	2038	967	5873	4432	32380	4474	31590	6268	64887	4291	59671
